# Hydrogen Sulfide Inhibited Sympathetic Activation in D-Galactose-Induced Aging Rats by Upregulating Klotho and Inhibiting Inflammation in the Paraventricular Nucleus

**DOI:** 10.3390/biomedicines11020566

**Published:** 2023-02-15

**Authors:** Hao Yu, Qiyao Yu, Yuan Mi, Ping Wang, Sheng Jin, Lin Xiao, Qi Guo, Yuming Wu

**Affiliations:** 1 Department of Physiology, Institute of Basic Medicine, Hebei Medical University, 361 Zhongshan East Road, Shijiazhuang 050017, China; 2 Department of Research, The Fourth Hospital of Hebei Medical University, Shijiazhuang 050011, China; 3 Department of Emergency, The Fourth Hospital of Hebei Medical University, Shijiazhuang 050011, China; 4 Experimental Center for Teaching, Hebei Medical University, Shijiazhuang 050017, China; 5 Hebei Collaborative Innovation Center for Cardio-Cerebrovascular Disease, Shijiazhuang 050017, China; 6 Key Laboratory of Vascular Medicine of Hebei Province, Shijiazhuang 050017, China

**Keywords:** hydrogen sulfide, sympathetic excitation, paraventricular nucleus, aging, klotho, inflammation

## Abstract

The present study aimed to explore the central relationship between cardiovascular conditions and aging. D-galactose (D-gal) was utilized to induce an accelerated aging model and to evaluate the effects of hydrogen sulfide (H_2_S) on aging-related cardiovascular risk factors and mechanisms. Eight-week-old Sprague Dawley rats were given an intraperitoneal injection of 250 mg/kg D-gal every day with or without H_2_S (56 μmol/kg) for 12 weeks. We found that D-gal treatment induced a noticeably aging-related increase in p16, p53 and p21 protein levels and senescence-associated beta-galactosidase staining. In addition, the level of noradrenalin was increased, accompanied by enhanced blood pressure and renal sympathetic nerve activity in aged rats. The greater sympathetic responses were related with the increased level of inflammation. The decreased level of klotho in the paraventricular nucleus neuron also contributed to sympathetic activation in D-gal-induced aged rats. However, the exogenous administration of H_2_S attenuated the sympathetic activity in aged rats, as evidenced by the decreased blood pressure, renal sympathetic nerve activity and noradrenalin level. The ameliorated cellular senescence, inflammation and heightened klotho in the paraventricular nucleus were attributed to the protective effects of H_2_S. The present study provides further evidence for the drug development of H_2_S for the prevention or treatment of the aging-associated cardiovascular diseases.

## 1. Introduction

Aging is a necessary biological process, defined as a progressive loss of physiological function, which affects most living beings [1]. During the aging process, the susceptibility to many diseases, including cancers, cardiovascular diseases, diabetes and neurodegenerative diseases, is increased in humans, leading to a gradual impact on the quality of life [2,3]. Aging is one of the independent risk factors for cardiovascular diseases, which increase as a function of age. Age-induced cardiovascular risk is closely related to autonomic nervous dysfunction, characterized by increased sympathetic and decreased parasympathetic activity, especially the continuous activation of sympathetic activity [4]. The imbalances in autonomic nervous system (ANS) functions are responsible for high blood pressure in older people [5,6]. Despite a large amount of literature regarding the association between age and cardiovascular diseases, the central mechanism involved in the occurrence and development of cardiovascular diseases needs further study.

The paraventricular nucleus (PVN) of the hypothalamus, one of the cardiovascular brain nucleuses, contains neurons that project directly to the intermediolateral cell column (IML) of the spinal cord, which innervates sympathetic preganglionic neurons and generates sympathetic nerve activity (SNA) to end organs via postganglionic neurons [7]. Increased levels of inflammatory factors in PVN, such as IL-1β, IL-6 and TNF-α, could lead to hypertension. Otherwise, inhibiting neuroinflammation in the PVN attenuates the plasma vasopressin level, kidney norepinephrine concentration and blood pressure (BP) [8]. Thus, neuroinflammation in the PVN represents a potential motive factor for aging-related changes that can contribute to the increase in blood pressure modulated by the ANS in older people.

Klotho, an anti-aging protein, is mainly expressed in the kidney and brain. Klotho knockout leads to accelerating aging and a shortening lifespan in models, whereas klotho overexpression slows down the progression of aging-related diseases and extends the lifespan in mice [9]. Its powerful aging-suppressing roles triggered a great interest and extensive research about its functions in age-related diseases. Previous studies showed that serum klotho levels were decreased in humans with aging [10] and with age-related diseases, such as cardiovascular diseases (including hypertension), chronic kidney disease, cancer or Alzheimer disease [11,12,13]. In addition, klotho is involved in various physiological pathways, such as the regulation of neuroinflammation, oxidative stress, angiotensin II and autophagy [14]. These findings provide convincing evidence that klotho may be a novel therapeutic target for age-related diseases.

Hydrogen sulfide (H_2_S), a novel gasotransmitter, widely exists in various tissues and organisms of the body and plays significant roles in a series of physiological and pathological conditions. Cystathionine-beta-synthase (CBS) and 3-mercapto-pyruvate sulfurtransferase (3-MST) are the major H_2_S-producing enzymes in the brain. Numerous studies have found that H_2_S inhibits inflammation and plays an important role in the progression of cardiovascular diseases [15], neurodegenerative diseases [16], respiratory disease [17], preeclampsia [18] and aging [19]. Moreover, treatment with sodium hydrosulfide (NaHS) increased the klotho level and effectively ameliorated renal tubulointerstitial fibrosis in a ureteral obstruction mouse model [20]. H_2_S affected the blood pressure by inhibiting sympathetic activation in our previous studies [21]. Given the above mentioned evidence, the interaction among H_2_S, klotho and sympathetic activation in PVN and their contribution to aging need to be explored.

In the present study, we determined whether sympathetic activation is a main characteristic of aged rats and explored whether H_2_S exerts a protective role on sympathetic activation in aged mice as well as the underlying mechanisms.

## 2. Materials and Methods

### 2.1. Preparation of the Animal Model

Eight-week-old male Sprague Dawley (SD) rats were obtained from the Animal Research Center of Hebei Medical University. The rats were housed in ordinary cages at room temperature (22 ± 2 °C), with 12 h light/dark cycles (lights on 7:00) with food and water ad libitum. All animal procedures were carried out according to the Animal Management Rule of the Ministry of Health, People’s Republic of China (documentation no. 55, 2001) and the Care and Use of Laboratory Animals published by the US National Institutes of Health (NIH Publication no. 85-23, revised in 1996) and approved by the Laboratory Animal Ethical and Welfare Committee of Hebei Medical University.

The rats were randomly assigned to three groups: the control group (0.9% saline, 1 mL/kg per day, i.p.), the D-gal-induced aging groups (D-gal, 250 mg/kg per day i.p.) and D-gal (250 mg/kg per day, i.p.) treated with NaHS (H_2_S donor, 56 μmol/kg per day i.p.) for 12 weeks. D-gal and NaHS were purchased from Sigma-Aldrich (Ltd., St. Louis, MO, USA). 0.9% saline was purchased from the Fourth Pharmaceutical Factory (Shijiazhuang, China).

### 2.2. Blood Pressure Measurement in Conscious Rats

In conscious rats, tail-cuff plethysmography (Chengdu Instrument Factory, Chengdu, Sichuan, China) was used to measure the systolic blood pressure (SBP) every 2 weeks from 8 to 20 weeks. Before the experiments, the rats were trained to accommodate to the procedures for the blood pressure measurement. First, each rat was placed on a heating pad for 15–20 min for tail artery vasodilatation before the measurement. Then, the rat was put in an appropriate recording chamber according to the body weight for 10 min to keep the rat comfortable and quiet. When the rats became quiet, the blood pressure measurement was always carried out between 15:00 and 18:00, and the final data were collected three to four times, on average.

### 2.3. Recording of Blood Pressure (BP) and Renal Sympathetic Nerve Activity (RSNA)

BP and RSNA were recorded as previously described [22]. Isoflurane (2% in O_2_) was used to anesthetize the rats. The trachea was cannulated for artificial ventilation by using a small animal ventilator (RWD407, Shenzhen, China) with isoflurane (2% in O_2_) during the experiment. The right femoral artery was intubated with a pressure transducer for monitoring BP. For the retroperitoneal exposure of the kidney, one branch of the left renal sympathetic nerve was separated near the renal vessel, placed on a pair of silver recording electrodes and immersed in warm (37 °C) mineral oil for potential recording. The PowerLab 15T data acquisition system (AD Instruments, QUAD, Bridge, Australia) was used to record BP and RSNA simultaneously. The integrating time of RSNA was 0.16 sec. At the end of the experiment, an overdose of sodium pentobarbital (200 mg/kg, i.v.) was used to obtain maximum RSNA and background electrical noise. The electrical noise levels were subtracted from the integrated RSNA values, and the percentage change in RSNA from the baseline was calculated as the percentage of Max [23].

### 2.4. PVN Microinjection

The anaesthetized rats were placed in a stereotaxic frame (RWD; Shenzhen, China) in a prone position. According to the Paxinos and Watson rat atlas, the microinjection point of PVN was 1.8 mm caudal from the bregma, 0.3 mm lateral to the midline and 7.8 mm below the skull surface. A glass micropipette connected to a microsyringe via a polyethylene tube was advanced into the PVN, and a microinjection pump was applied for delivering Angiotensin II (Ang II). Blood pressure and RSNA were recorded simultaneously by using Powerlab (AD Instruments, Sydney, Australia).

### 2.5. Measurements of Plasma, Cerebrospinal Fluid Norepinephrine (NE) and IL-1β

At the end of 20 weeks, the rats were sacrificed for collecting blood from the venae cava inferior and cerebrospinal fluid. The approach was used to test the levels of NE and IL-1β following the instructions of the ELISA kits (COIBO biotechnology, Shanghai, China). The final solution was read at a 450 nm wavelength on a microplate reader (Powerwave XS2, BioTek, Winooski, VT, USA).

### 2.6. Western Blotting Analysis

As previously described [21,24], the fresh brain block containing PVN was dissected and fixed on a vibratome (VT1200S, Leica, Wetzlar, Germany), which was used to cut the brain slices. PVN pieces that were 250 μm thick were punched out bilaterally using fine-tip forceps under a microscope (EZ4, Leica, Wetzlar, Germany) for further research. The PVN pieces were placed on the ice and cracked by an ultrasound with lysates. Then, the specimens were centrifuged at 12,000 rpm for 15 min at 4 °C to obtain the supernatant for the protein assay by the Bradford assay (Generay Biotechnology, Shanghai, China). Then, 35 μg protein samples were loaded in each lane and separated by electrophoresis. After the electrophoresis on 12% SDS-PAGE gel, the protein was transferred onto polyvinylidene fluoride membranes (Millipore Corp, Burlington, MA, USA). The polyvinylidene fluoride membranes containing protein were blocked with 0.1% Tween-20 tris-buffered saline containing nonfat milk (5%) for 90 min at room temperature. After being blocked, the membranes were incubated at 4 °C overnight with the primary antibodies for NLRP3 (1:1000, 19771-1-AP, Proteintech, Wuhan, China), Caspase-1 (1:1000, ab1872, Abcam, Waltham, MA, USA), IL-1β (1:1000, 16806-1-AP, Proteintech, Wuhan, China), IL-10 (1:1000, ab9969, Abcam, Waltham, MA, USA), Klotho (1:1000, ab203576 Abcam, Waltham, MA, USA), 3-mercaptopyruvate sulfurtransferase (3-MST, 1:1000, ab85211, Abcam, Waltham, MA, USA), cystathionine β-synthase (CBS, 1:2000, 14787-1-AP, Proteintech, Wuhan, China), p16 (1:1000, ET1608-62, HUABIO, Hangzhou, China), p21 (1:1000, ER1914-57, HUABIO, Hangzhou, China) and p53 (1:1000, 21891-1-AP, Proteintech, Wuhan, China). Western blotting reagents (Millipore Corp., Burlington, MA, USA) were used to detect signals, and blots were exposed to an X-ray film for densitometric analysis. The protein intensity was normalized to that of GAPDH (1:5000, 10494-1-AP, Proteintech, Wuhan, China).

### 2.7. Senescence-Associated β-Galactosidase (SA-β-Gal) Staining and Immunofluorescence Staining

The rats were anesthetized with sodium pentobarbital (50 mg/kg, i.p.) and then perfused transcardially with physiological saline (0.9% NaCl), followed by 4% para-formaldehyde (PFA). The brains containing PVN were dissected and fixed in 4% PFA overnight and then incubated in 30% sucrose phosphate buffer at 4 °C until they sank to the bottom. Coronal sections (25 μm) were cut on a freezing microtome (CM1950, Leica, Wetzlar, Germany). For dual immunohistochemistry, the brain sections were washed in PBS three times. Then, the slices were incubated overnight with primary antibodies in PBS containing 0.25% Triton X-100 (PBST) at 4 °C: anti-rabbit klotho (1:250, ab203576, Abcam); anti-mouse neuronal nuclei (NeuN) (1:200, 66836-1-lg, Proteintech); anti-mouse ionized calcium binding adapter molecule 1(Iba1) (1:250, GTX632426, GeneTex, Irvine, CA, USA) and anti-mouse glial fibrillary acidic protein (GFAP) (1:50, ab4648, Abcam) for 12 h. The slices were washed three times with PBS before the incubation with secondary antibodies at room temperature for 2 h: Alexa 647-conjugated goat anti-rabbit (1:500, ab150083, Abcam) and Alexa 488-conjugated goat anti-mouse (1:500, ab150117, Abcam). Finally, the brain slices were mounted on glass slides, dried, dehydrated and cover-slipped. Fluorescent images were obtained by a microscope (DM6 B Thunder imager, Leica, Wetzlar, Germany).

SA-β-gal staining involved using a senescence β-galactosidase staining kit (Beyotime Institute of Biotechnology, Shanghai, China). Briefly, according to the procedure of the kit, the brain slices (25 μm) were washed with PBS three times and then incubated for 12 h away from light in SA-β-gal staining solution at 37 °C. Then, the slices were washed with PBS and cover-slipped for direct imaging with a microscope (DM6 B Thunder imager, Leica, Wetzlar, Germany)

### 2.8. Statistical Analysis

All data are expressed as the means ± SD. Prism version 5.0 (GraphPad Software Inc., San Diego, CA, USA) was used for the analysis. One-way or two-way ANOVA was used to compare the differences among groups. Student-Newman–Keuls and Bonferroni tests were used for further analysis. *p* < 0.05 was considered statistically significant.

## 3. Results

### 3.1. Effect of H_2_S on SA-β-Gal Activity and Senescence-Associated Protein Level in PVN

**Figure 1:**
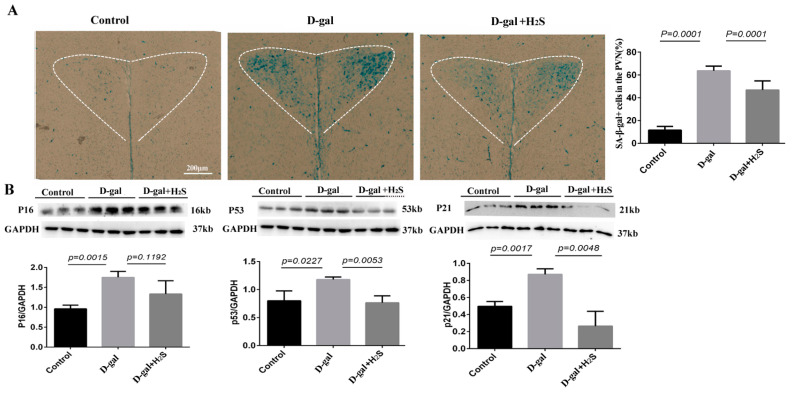
Effect of hydrogen sulfide (H_2_S) on the senescence-associated β-galactosidase (SA-β-gal) activity and the senescence-associated protein level in the paraventricular nucleus (PVN). (**A**) SA-β-gal staining showed the level of SA-β-gal positive cells, colored blue, in the PVN with the control and 12 weeks of D-gal treatment with or without H_2_S groups; *n* = 3, scale bars = 200 μm. (**B**) Representative western blots analysis of the protein levels of p53, p16 and p21 extracted from the PVN of the control and those undergoing 12 weeks of D-gal treatment with or without H_2_S; *n* = 3.

We established an accelerated aging model involving treatment with D-gal for 12 weeks. Then, we detected the most widely recognized aging biomarkers, the SA-β-gal activity and the senescence-associated protein level, in the PVN. D-gal treatment greatly increased SA-β-gal positive cells (colored blue in the images, Figure 1A) in the PVN. The senescence-associated protein levels of P16, P21 and P53 were also increased. All of these findings confirmed the aging changes of the brain in D-gal-induced aging rats. However, supplementation with H_2_S effectively inhibited SA-β-gal positive cells and the senescence-associated protein level in the PVN, which suggests an anti-aging effect of H_2_S. The above results showed that H_2_S could effectively attenuate D-gal-induced aging in rats.

### 3.2. The Systolic Blood Pressure (SBP) in Conscious Rats and the Plasma and Cerebrospinal Fluid NE Level

**Figure 2:**
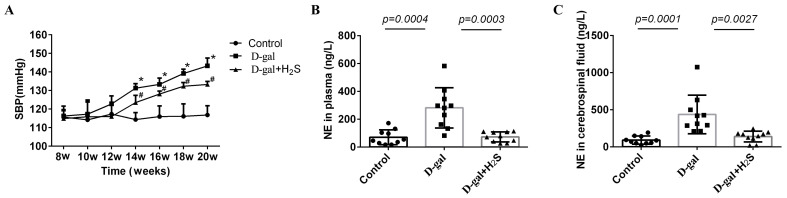
(**A**) Time course of systolic blood pressure (SBP) in conscious rats from 8 to 20 weeks in the control and in D-gal rats alone and with H_2_S groups. * *p* < 0.05 vs. control, ^#^ *p* < 0.05 vs. D-gal rats, *n* = 6. (**B**) Serum NE level in rats treated with D-gal alone and with H_2_S for 12 weeks, *n* = 10. (**C**) Cerebrospinal fluid NE level of rats treated with D-gal alone and with H_2_S for 12 weeks, *n* = 10. Data are the means ± SD. (**A**), Two-way ANOVA. (**B**,**C**) One-way ANOVA.

SBP was higher in aging rats than in the control from 14 to 20 weeks after treatment with D-gal (14 weeks, 131.28 ± 2.41 vs. 114.35 ± 3.45 mmHg; 16 weeks, 133.32 ± 3.42 vs. 115.97 ± 5.26 mmHg; 18 weeks, 139.18 ± 2.37 vs. 116.15 ± 6.13 mmHg; 20 weeks, 143.27 ± 4.24 vs. 116.85 ± 4.56 mmHg, *p* < 0.05) (Figure 2A). The level of NE, considered as a marker of sympathetic activation, was increased in plasma and cerebrospinal fluid. The level of NE was increased in D-gal-induced aging groups (Figure 2B,C). When the D-gal-induced aging rats were treated with H_2_S, the level of NE was decreased (plasma, 72.78 ± 35.76 vs. 281.56 ± 145.07 ng/L; cerebrospinal fluid, 139.05 ± 72.90 vs. 436.97 ± 260.37 ng/L), and SBP was significantly decreased (14 weeks, 123.53 ± 3.92 vs. 131.28 ± 2.41 mmHg; 16 weeks, 128.15 ± 1.57 vs. 133.32 ± 3.42 mmHg; 18 weeks, 132.32 ± 2.03 vs. 139.18 ± 2.37 mmHg; 20 weeks, 133.28 ± 1.67 vs. 143.27 ± 4.24 mmHg, *p* < 0.05).

### 3.3. The Effect of H_2_S on Basal RSNA, BP and Ang II-Induced Changes in RSNA and BP in D-Gal-Induced Aging Rats

**Figure 3:**
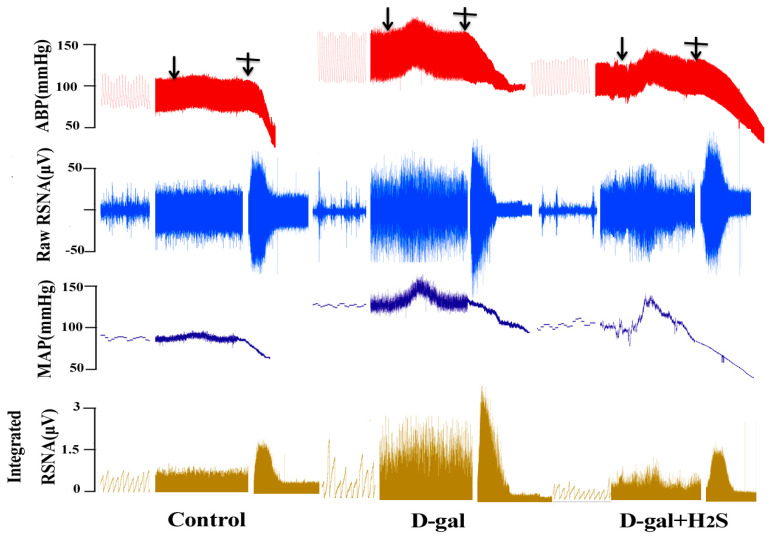
Effect of H_2_S on basal renal sympathetic nerve activity (RSNA), arterial blood pressure (ABP) and Ang II-induced changes in RSNA and ABP in D-gal-induced aging rats. Raw trace recordings showing that the microinjection of Ang II (10^−5^ mmol/L, 100 nL) into the PVN of rats increased the mean arterial pressure (MAP) and RSNA in male rats from the control, D-gal and D-gal + H_2_S groups, *n* = 6. “
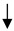
” means microinjection of Ang II; “
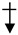
” means induced maximum RSNA.

**Figure 4:**
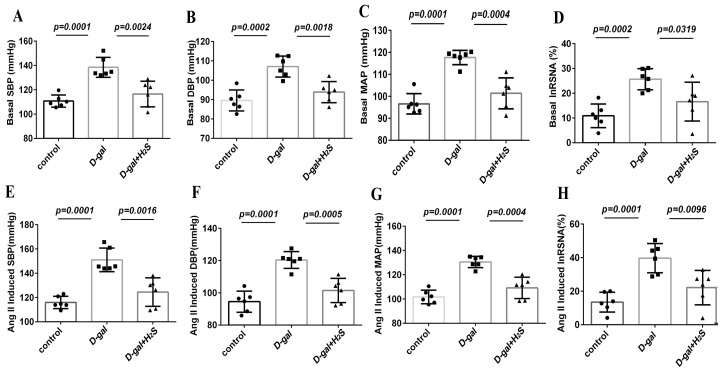
Effect of H_2_S on basal RSNA, blood pressure (BP) and Ang II-induced changes in RSNA and BP in D-gal-induced aging rats. Summary data showing the baseline and peak effect of Ang II (10^−5^ mmol/L, 100 nL) microinjected into the PVN on SBP, diastolic blood pressure (DBP), MAP and RSNA %Max. (**A**–**D**) showed the summary data of basal SBP, DBP, MAP and RSNA %Max. (**E**–**H**) showed the summary data of Ang II-induced SBP, DBP, MAP and RSNA %Max. Data are the mean ± SD, *n* = 6, one-way ANOVA.

The basal sympathetic outflow was evaluated by recording RSNA and BP. Figure 3 and Figure 4 show an original trace and the summary data of the sympathetic responses to Ang II (100 nL, 10^−5^ mmol/L) microinjected into the PVN of D-gal-induced aging rats with or without H_2_S. Basal RSNA and BP were increased in aging rats as compared with controls (SBP, 138.48 ± 8.17 vs. 110.76 ± 5.01; diastolic blood pressure (DBP), 107.10 ± 5.4 vs. 89.55 ± 5.41; mean arterial pressure (MAP), 117.65 ± 3.25 vs. 96.62 ± 4.64 mmHg; RSNA, 25.68 ± 4.27% vs. 10.90 ± 4.78% Max, *p* < 0.05). The microinjection of Ang II in the PVN significantly enhanced RSNA and BP in aging versus control rats (SBP, 151.08 ± 9.70 vs. 116.05 ± 5.08; DBP, 120.39 ± 5.20 vs. 94.58 ± 6.55; MAP, 130.56 ± 4.69 vs. 101.74 ± 5.55 mmHg; RSNA, 39.67 ± 8.69% vs. 13.52 ± 5.93% Max, *p* < 0.05). As compared with aging rats, those receiving H_2_S showed attenuated sympathoexcitatory responses to Ang II (SBP, 124.49 ± 11.72 vs. 151.08 ± 9.7; DBP, 101.50 ± 7.54 vs. 120.39 ± 5.20; MAP, 109.17 ± 8.82 vs. 130.56 ± 4.69 mmHg; RSNA, 22.17 ± 10.22% vs. 39.67 ± 8.69% Max, *p* < 0.05).

### 3.4. Inflammation-Related Protein Level in the PVN and IL-1β Level in Plasma and Cerebrospinal Fluid

**Figure 5:**
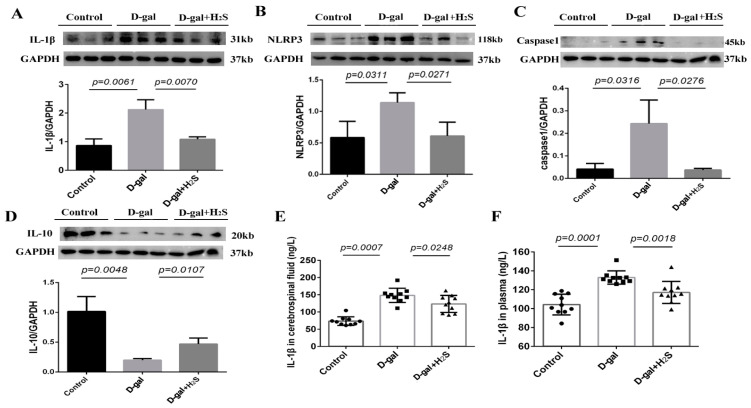
(**A**–**D**) Protein levels of NLRP3, caspase-1, IL-1β and IL-10 in the PVN of the control, D-gal and D-gal + H_2_S groups. *n* = 3. GAPDH was used for normalization. (**E**,**F**) Level of IL-1β in the cerebrospinal fluid and plasma in the rat groups. Data are the mean ± SD, *n* = 10, one-way ANOVA.

The protein levels of IL-1β, NLRP3 and caspase-1 were significantly increased in D-gal-induced aging rats, whereas that of IL-10 was decreased in the PVN in aging rats. The exogenous administration of hydrogen sulfide significantly reversed the above protein levels. The plasma and cerebrospinal fluid level of IL-1β was increased in aging rats (plasma, 132.84 ± 7.00 vs. 104.28 ± 10.96 ng/L; cerebrospinal fluid, 148.20 ± 20.64 vs. 73.69 ± 12.54 ng/L) and was decreased with H_2_S treatment (plasma, 117.11 ± 11.70 vs. 132.84 ± 7.00 ng/L; cerebrospinal fluid, 123.31 ± 24.62 vs. 148.20 ± 20.64 ng/L) (Figure 5).

### 3.5. Effect of the Exogenous Administration of H_2_S on the Levels of Klotho and Endogenous H_2_S Enzymes

**Figure 6:**
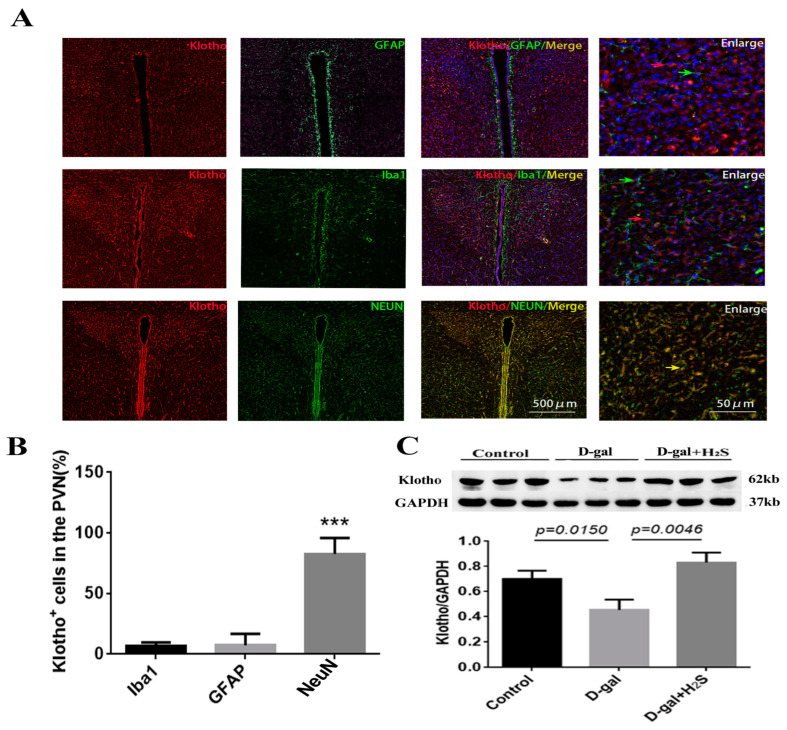
Effects of the exogenous administration of H_2_S on the level of klotho. (**A**) Immunofluorescence staining of klotho (red) and neuronal nuclei (NeuN)/glial fibrillary acidic protein (GFAP)/ionized calcium binding adapter molecule 1 (Iba1) (green) in the PVN from 20-week-old male rats. (**B**) Summary data of the percentage of klotho positive cells in NeuN/GFAP/Iba1, counted from five consecutive visual fields in one slide and two slides from one rat. *n* = 3. *** *p* < 0.001 vs. Iba1 or GFAP (**C**) Protein level of klotho in the PVN from the control, D-gal and D-gal + H_2_S groups. Data are the mean ± SD, *n* = 3. GAPDH was used for normalization. One-way ANOVA.

**Figure 7:**
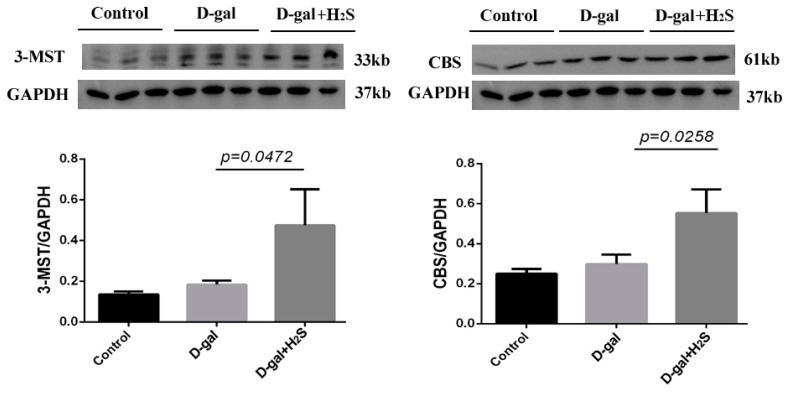
Effect of the exogenous administration of H_2_S on the level of endogenous H_2_S enzymes. Protein levels of cystathionine β-synthase (CBS) and 3-mercapto-pyruvate sulfurtransferase (3-MST) in the PVN from the control, D-gal and D-gal + H_2_S groups. Data are the mean ± SD, *n* = 3. One-way ANOVA. GAPDH was used for normalization.

Immunofluorescence staining showed klotho mainly expressed in neurons. The protein level of klotho was significantly decreased in the PVN of aging rats; exogenous treatment with hydrogen sulfide could increase the level of klotho in the PVN (Figure 6). We also checked the levels of CBS and 3-MST, endogenous enzymes of H_2_S, in D-gal-induced aging rats alone and with H_2_S. The levels of the endogenous enzymes of H_2_S were not significantly changed in aging rats (Figure 7). However, treatment with H_2_S significantly increased their protein levels.

## 4. Discussion

In the present study, conscious SBP and sympathetic outflow were increased in aging rats. The acute infusion of Ang II into the PVN induced a greater increase in BP and RSNA in the D-gal group than it did in the control. The senescence-associated protein level and SA-β-gal staining were markedly increased in aging rats. The inflammation level in the PVN and plasma was increased and the anti-aging protein klotho level was decreased in aging animals. Although the levels of endogenous enzymes of H_2_S were not changed in aging rats, treatment with H_2_S significantly increased the protein levels of these enzymes and upregulated klotho, for a protective role in anti-aging.

Classically, aging animal models can be divided into naturally aging models and accelerated aging models. Accelerated aging models are induced in a shorter time, and animals have a higher survival rate during the experiment, whereas the naturally aging model will take much more time and expense, and it has a higher mortality. Therefore, a D-gal-induced mimetic aging model is one of the most preferred accelerated aging models; it has the fewest side effects and a higher survival rate during the experimental period [25]. D-gal is being used more to study aging. In vivo and in vitro treatment with D-gal could reduce longevity in animals and cultured cells [26] and lead to cognitive dysfunction [27], neurodegeneration [28], cardiovascular disease [29] and immune system dysfunction [30]. Moreover, D-gal-induced accelerated aging models can increase aging markers such as advanced glycation end-products, senescence-associated genes and senescence associated beta-galactosidase (SA-β-gal) staining [31,32,33]. Our results also show that D-gal-induced aging in rats increased the levels of senescence-associated protein and SA-β-gal staining in the PVN, so our experiment successfully established a mimetic aging model induced by D-gal. Consistent with the previous reports, these D-gal-treated animals are suitable for studying the mechanism of aging. We also found that H_2_S inhibited D-gal-induced neuronal senescence, as evidenced by the decrease in SA-β-gal positive cells and the downregulation of senescence-associated protein levels in the PVN. Neuronal senescence in PVN may be involved in aging-induced sympathetic activation.

Sympathetic nervous system activation is one of the main pathophysiologic mechanisms associated with worse outcomes in some cardiovascular diseases [34]. Increasing renal sympathetic nerve activity indicates an overall sympathetic activation in cardiovascular diseases. In the present experiment, we recorded RSNA to reflect sympathetic nerve activity in aging rats. Ang II, in addition to its classical and well-known hemodynamic action, has increased sympathetic activity effects. Additionally, Ang II is one of the main factors leading to cardiovascular disease. There have been a number of studies on Ang II and sympathetic activity in rodents [35,36]. Our previous study reported that the intracerebroventricular administration of Ang II induced greater effects of sympathetic outflow in the offspring of hypertensive rats, which can easily develop into hypertension [22]. The present results show that BP and RSNA were steadily increased in aging rats, and the rats exhibited greater sympathetic responses to Ang II microinjection in the PVN. These phenomena imply that aging rats can easily exhibit hypertension and that aging is a major risk factor for cardiovascular disease. Moreover, the level of NE in plasma and cerebrospinal fluid was increased in D-gal-induced aging rats. However, elevated circulating levels of NE imply the activation of the sympathetic nervous system in the etiology of cardiovascular diseases [37]. Thus, the effects of D-gal-induced aging on sympathetic activation could be a potential risk factor of cardiovascular diseases. Previous studies suggested that H_2_S had sympathetic inhibition effects [38]. BP and RSNA were effectively reverted in H_2_S-treated groups, so H_2_S could potentially regulate and maintain the autonomic nerve system homeostasis by reducing neuron cellular senescence in the PVN.

Chronic low-grade sterile inflammation during aging, also known as “inflammaging”, is a hallmark of aging [39,40]. Accumulating studies have shown that inflammaging is a potential risk factor reducing tissue repair and generative capacity, and it is an important contributing factor to many age-associated diseases [41,42]. We found increased levels of the inflammation-related proteins IL-1β, NLRP3 and caspase-1, whereas that of IL-10 was decreased in the PVN in D-gal-induced aging rats. The plasma and cerebrospinal fluid levels of IL-1β were also increased. We also found senescent cells in the PVN by SA-β-gal staining in aging rats. Senescent cells accumulate in aged tissues and can trigger age-associated inflammation [40,43]. However, neuron inflammation can induce sympathetic activation, which is a potential risk factor for the occurrence of cardiovascular diseases [44]. Thus, senescence-induced inflammation might contribute to sympathetic activation and bridge the gap between aging and cardiovascular disease.

The level of klotho, an anti-aging protein, decreases with aging and aging-related diseases such as cardiovascular disease, Alzheimer disease, kidney disease, chronic obstructive pulmonary disease and cerebrovascular diseases. The downregulation of klotho promotes the progression of these diseases; conversely, the overexpression of klotho can have therapeutic effects on age and aging-related diseases [45,46,47]. The histological and molecular biology assessment we performed showed that klotho was mainly expressed in neurons in the PVN, and its level was decreased in D-gal-induced aging rats. When the aging rats were treated with H_2_S, the level of klotho was increased significantly. Therefore, the protective effect of H_2_S on D-gal-induced aging in rats may enhance the levels of the anti-aging proteins.

However, we found that the levels of CBS and 3-MST, mainly endogenous H_2_S-synthesizing enzymes in the brain, were not decreased in the PVN of D-gal rats, most likely due to compensation. Additionally, after 12 weeks of the intraperitoneal injection of NaHS (56 μmol/kg/day), the levels of CBS and 3-MST were increased in the PVN of D-gal-induced aging rats. Our results suggested that the exogenous administration of NaHS influenced the expression of endogenous H_2_S-synthesizing enzymes in the PVN of D-gal-induced aging rats. However, the mechanism needs to be further studied. An amount of 50 mg/kg D-gal with or without NaHS (50 and 100 μmol/kg/day) daily for 2 months led to a lower CSE level and unchanged 3-MST and CBS levels in the hearts of mice treated with D-gal and increased CSE and CBS levels, but not the 3-MST level, in mice treated with D-gal and NaHS. In liver tissues, D-gal failed to influence the levels of the three H_2_S-producing enzymes. However, sustained 50 μmol/kg/day of NaHS further increased CSE and CBS levels. In the kidney, only CSE expression was decreased upon D-gal exposure; NaHS supply only increased CSE and CBS levels. In the human umbilical vein endothelial cells, D-gal treatment did not influence the levels of the three H_2_S-producing enzymes, however, NaHS improved CSE and CBS levels but failed to alter the 3-MST level [48]. In addition, 2-week NaHS (25–100 μmol/kg/day) treatment increased the levels of CSE, CBS and 3-MST in the ischemic myocardium after myocardium infraction [49]. Our previous study found that exogenous H_2_S enhanced the levels of CSE and 3-MST, but not the CBS level, in the myocardial tissue of aging rats [50]. Another similar study found that the level of CSE increased and that of CBS not improved in myocardium infraction after drinking H_2_S-releasing solution for 4 weeks [51]. The possible reasons for the different results could be the tissue specificity and the duration and doses of NaHS and D-gal treatment. Overall, NaHS treatment may increase the activity of CSE/CBS/3-MST to produce more endogenous H_2_S, which has persistent protective effects and fights against aging.

There are some limitations to our present study. We need further investigations of how H_2_S regulates klotho in neurons and how it causes signaling to neurons in the PVN. Whether the cardiovascular characteristics in the naturally aging model are consistent with those in the accelerated model is unknown. We are now trying to study the above-mentioned problems in the next part of our experiment.

## 5. Conclusions

D-gal-induced aging rats can show changes in the klotho level and the secretion of inflammatory factors in the PVN, resulting in autonomic dysfunction and hypertension. Treatment with H_2_S could prevent the sympathetic activation, which may be related to the enhanced klotho level and the attenuated senescence cell-activated inflammatory mediators in the PVN of aging rats. These results suggest that H_2_S plays important roles in aging. Methods for modulating H_2_S may be a promising treatment strategy for combatting aging and related cardiovascular disease.

## Data Availability

The data presented in this study is available upon reasonable request from the corresponding author.
